# David Walsh and Gerry McCartney. Social Murder? Austerity and Life Expectancy in the UK

**DOI:** 10.1093/eurpub/ckaf011

**Published:** 2025-02-13

**Authors:** Ted Schrecker

**Affiliations:** Population Health Sciences Institute, Newcastle University, Newcastle upon Tyne, United Kingdom

Richard Horton, editor-in-chief of *The Lancet*, wrote in 2017 that: ‘Austerity is the calling card of neoliberalism. Its effects follow an inverse harm law—the impact of increasing amounts of austerity varies inversely with the ability of communities to protect themselves. Austerity is an instrument of malice. …. What is promoted as fiscal discipline is a political choice. A political choice that deepens the already open and bloody wounds of the poor and precarious’. In this book, which adopts the concept of social murder advanced in Engels’ *The Condition of the Working Class in England* (1845), Glasgow-based population health researchers David Walsh and Gerry McCartney offer a formidable compilation of evidence for Horton’s perspective as it applies to the UK, providing a multitude of graphs and charts as well as seven authenticated and heartbreaking vignettes of austerity’s casualties.

Their focus is on the two primary elements of post-2010 austerity: cuts to local authority budgets, and therefore to the services those authorities provide; and cuts in benefits provided directly to the more than 9 million low-income households in Great Britain, who ‘have received either less help, or no help at all’. Each element has disproportionately worsened the situations of the poorest people, and the poorest places. Among the most conspicuous and extreme manifestations were a rise in food insecurity (the number of food banks in England rose from 35 in 2010/11 to 1300 at the end of the decade) and homelessness. The authors make a compelling case for a causal connection with the fact that by 2012, life expectancy across the population had flatlined, and ‘in the poorer areas of the UK, in a complete reversal of previous long-term trends, people have been *dying younger*, and dying in greater numbers than before’—a pattern that developed well before the coronavirus-19 (COVID-19) pandemic. Chapter 5 is a vital, stinging description of the contortions engaged in by official actors trying to avoid critiquing austerity, making the often-neglected point that ‘wildly different thresholds for evidence quality are used to explain different public health problems’; the chapter that follows describes how and why the pandemic magnified austerity-related health inequalities.

Austerity did not just happen. It must be understood with reference to the rising tide of neoliberalism as an organizing principle of public policy, a decades-long (and well-funded) process dating back at least to the founding of the Mont Pèlerin society in 1947, aimed at what anthropologist and geographer David Harvey describes as ‘the restoration of class power’. Against this background, austerity’s upward redistribution of income, wealth, and associated political influence emerges as a feature, not a bug. Along with the expanding social science literature that explains these points, Walsh and McCartney’s superlative book should occupy a central place on undergraduate and postgraduate public health module reading lists.

